# Alteration of Whole Brain ALFF/fALFF and Degree Centrality in Adolescents With Depression and Suicidal Ideation After Electroconvulsive Therapy: A Resting-State fMRI Study

**DOI:** 10.3389/fnhum.2021.762343

**Published:** 2021-11-11

**Authors:** Xiao Li, Renqiang Yu, Qian Huang, Xiaolu Chen, Ming Ai, Yi Zhou, Linqi Dai, Xiaoyue Qin, Li Kuang

**Affiliations:** ^1^Department of Psychiatry, The First Affiliated Hospital of Chongqing Medical University, Chongqing, China; ^2^Department of Radiology, The First Affiliated Hospital of Chongqing Medical University, Chongqing, China; ^3^The First Branch, The First Affiliated Hospital of Chongqing Medical University, Chongqing, China; ^4^Department of the First Clinical Medicine, Chongqing Medical University, Chongqing, China

**Keywords:** MDD, adolescent, ALFF, degree centrality, resting-state fMRI, suicidal ideation, electroconvulsive therapy

## Abstract

Major depressive disorder (MDD) is one of the most widespread mental disorders and can result in suicide. Suicidal ideation (SI) is strongly predictive of death by suicide, and electroconvulsive therapy (ECT) is effective for MDD, especially in patients with SI. In the present study, we aimed to determine differences in resting-state functional magnetic resonance imaging (rs-fMRI) in 14 adolescents aged 12–17 with MDD and SI at baseline and after ECT. All participants were administered the Hamilton Depression Scale (HAMD) and Beck Scale for Suicide Ideation (BSSI) and received rs-fMRI scans at baseline and after ECT. Following ECT, the amplitude of low frequency fluctuation (ALFF) and fractional ALFF (fALFF) significantly decreased in the right precentral gyrus, and the degree centrality (DC) decreased in the left triangular part of the inferior frontal gyrus and increased in the left hippocampus. There were significant negative correlations between the change of HAMD (ΔHAMD) and ALFF in the right precentral gyrus at baseline, and between the change of BSSI and the change of fALFF in the right precentral gyrus. The ΔHAMD was positively correlated with the DC value of the left hippocampus at baseline. We suggest that these brain regions may be indicators of response to ECT in adolescents with MDD and SI.

## Introduction

Suicide is an important global health concern cited as the 20th leading cause of death worldwide. Major depressive disorder (MDD) is a major risk factor for suicide (Kessler et al., 2005); previous research has reported that approximately 15% of patients with MDD die by suicide (Chen and Dilsaver, 1996; Angst et al., 2013). Moreover, suicide in adolescents has become a severe public health and social dilemma. A 2013 survey of thousands of teenagers found that one in eight demonstrated suicidal ideation (SI; Nock et al., 2013). SI is defined as “thoughts about death, dying, plans for suicide, or desire for death” (Miller et al., 2018; Levi-Belz et al., 2019), it is strongly predictive of death by suicide (Klonsky et al., 2016). Additionally, MDD with SI is related to higher rates of poor treatment response (Szanto et al., 2003), and is thought to have different neuropsychological correlates compared to MDD without SI (Marzuk et al., 2005). Therefore, measuring SI in patients with MDD is necessary and may help determine the risk of suicide.

There are many challenges in the treatment of MDD in adolescents. Importantly, adolescents do not exhibit the same symptoms as adults, resulting in difficulties in diagnosis (Lee et al., 2019). Some adolescents with depressive symptoms develop bipolar disorder (BD; Egeland et al., 2000), comorbid borderline personality disorder (Horesh et al., 2003), SI, or non-suicidal self-injury (NSSI; Huang et al., 2021); all of which increase the difficulty of treatment.

Electroconvulsive therapy (ECT) has been found to be effective in schizophrenia, depression, and eating disorders (Pagnin et al., 2004; Sanghani et al., 2018; Pacilio et al., 2019). For different psychiatric illnesses or age groups with SI, ECT can be an effective and appropriate treatment option (Ghaziuddin et al., 2020; Meyer et al., 2020). ECT is also considered a treatment option for adolescents with MDD, especially for adolescents with MDD and SI or related behaviors (Puffer et al., 2016; Mitchell et al., 2018). A previous study has found that adolescents with mood disorders who were administered ECT demonstrated a reduction in SI and NSSI (Ghaziuddin et al., 2020).

MRI is widely used to evaluate brain change in MDD patients after ECT, Wilkinson et al. (2017) found ECT induced hippocampal volume changes in MDD patients, a systematic review of fMRI showed the amplitude of low frequency fluctuations (ALFF) changed widely across the brain, such as orbital gyrus, inferior frontal gyrus, precentral gyrus, etc. (Porta-Casteràs et al., 2020). ALFF is an rs-fMRI-derived measure that reflects the magnitude of spontaneous blood-oxygen-level-dependent (BOLD) signal (Nugent et al., 2015). The fractional ALFF (fALFF) is one of the most common metrics used to quantify these oscillations (Zou et al., 2008); however, both have been used to infer brain activity in psychiatric disorders with or without suicidal behaviors (Bu et al., 2019; Lan et al., 2019; Zhang et al., 2020). One recent study investigated ALFF in depressed patients with SI and found higher ALFF values in the right hippocampus and bilateral thalamus and caudate compared to patients without SI (Lan et al., 2019). In addition, Liu et al. (2015) found that a reduction in depressive symptoms was negatively correlated with increased ALFF in the left hippocampus after eight ECT sessions. Degree centrality (DC), which focuses on the relation of a voxel with the connectivity of the entire network (Buckner et al., 2009), can also be used to measure brain function. Gao et al. (2016) found compared with controls, depressive subjects showed decreased DC in the right parahippocampal gyrus, and elevated DC in the left inferior frontal gyrus.

In the present study, we examined whole brain ALFF/fALFF and DC among adolescents with MDD and SI. We hypothesized that: (1) ECT would make ALFF/fALFF and DC changes in adolescents MDD with SI; (2) ECT-induced brain function changes may be the treatment mechanism for MDD with SI.

## Materials and Methods

### Participants

The present study included 14 adolescents with MDD and SI aged 12–17 years. The participants were recruited from the inpatient clinics at the Department of Psychiatry, First Affiliated Hospital of Chongqing Medical University, China. The presence or absence of diagnoses was independently determined by two experienced psychiatrists using the Mini International Neuropsychiatric Interview for Children and Adolescents (MINI-KID).

Clinical symptoms were assessed with the 17-item Hamilton Depression Rating Scale (HAMD-17; Hamilton, 1960). The Chinese version of the instrument has been found to be reliable and valid (Zhao and Zheng, 1992). SI intensity was assessed with the Beck Scale for Suicide Ideation (BSSI; Beck et al., 1979). The BSSI is a 19-item self-report measure designed to assess the current attitude, behaviors, and plan to commit suicide. All items are rated on a 3-point scale of intensity and generate a total score from 0 to 38. The results of the BSSI were confirmed by two psychiatrists through a clinical interview. The Chinese version of the BSSI shows acceptable reliability and validity (Li et al., 2010).

Participants were excluded if they: (1) had a neurological or serious physical condition, any history of alcohol or drug abuse, any other somatic diseases, or morphological anomalies of the brain; (2) had any surgically placed electronic or metal materials that might interfere with fMRI assessment; (3) took medications in recent five drug half-life; or (4) had head motion exceeding 3 mm in translation or 3° in rotation.

The present study protocol was approved by the Human Research and Ethics Committee of the First Affiliated Hospital of Chongqing Medical University (no. 2017-157). Written informed consent was obtained from all adolescents and their caregivers.

### Electroconvulsive Therapy

All patients underwent modified bi-fronto-temporal ECT that was conducted using a Thymatron DGx (Somatics, LLC, Lake Bluff, IL, USA) at the First Affiliated Hospital of Chongqing Medical University (Du et al., 2016). The first three courses of ECT took place on continuous days; the remaining courses of ECT were performed every 2 days, with a break on weekends. After 2 weeks, the ECT was complete. The first energy for ECT was determined according to the patient’s age: energy percent = age × 0.5%. The stimulation energy was adjusted based on the seizure time. The energy was increased by 5% in the subsequent treatment if the seizure time was <25 s. Anesthesia was induced with succinylcholine (0.5–1 mg/kg) and diprivan (1.5–2 mg/kg). All the patients received antidepressants, with sertraline (*n* = 9, 64.3%), fluoxetine (*n* = 5, 35.7%). Ten patients received antipsychotics, with quetiapine (*n* = 4, 28.6%), olanzapine (*n* = 4, 28.6%), aripiprazole (*n* = 2, 14.3%). Two patients received propranolol (*n* = 2; 29.5%).

### Acquisition of rs-fMRI Data

MR images were obtained using a 3T GE Signa HDxt scanner (General Electric Healthcare, Chicago, IL, USA) with an 8-channel head coil. Participants were instructed to relax with their eyes closed, stay awake, and avoid thinking as much as possible. None of the patients reported falling asleep during the scan. Foam pads and earplugs were used to fix their heads to minimize head motion and reduce machine noise, respectively. The echo-planar imaging pulse sequence parameters were as follows: repetition time (TR) = 2,000 ms; echo time (TE) = 40 ms; field of view (FOV) = 240 × 240 mm^2^; matrix = 64 × 64; flip angle = 90°; slice number = 33; slice thickness/gap = 4.0/0 mm; scanner time = 8 min; and 240 volumes. Three-dimensional T1-weighted MR images were used for rs-fMRI co-registration *TR* = 24 ms; *TE* = 9 ms; *FOV* = 240 × 240 mm^2^; matrix = 256 × 256; flip angle = 90°; and slice thickness/gap = 1.0/0 mm.

### Image Preprocessing

All data preprocessing was performed in MATLAB (MathWorks, Natick, MA, USA) using DPARSF (version 4.3; Data Processing Assistant for Resting-State fMRI^1^), which is based on SPM12^2^. The first five time points were discarded to allow for signal equilibration. Images were then corrected for slice timing and head motion. Functional images were spatially normalized to the Montreal Neurological Institute space and resampled at 3 × 3 × 3 mm^3^. Nuisance regression was performed using the 24 head motion parameters, white matter, and cerebrospinal fluid signals as covariates. Linear trends were removed. Finally, the images were bandpass filtered (0.01–0.1 Hz) to reduce low-frequency drift and high-frequency physiological noise.

### Calculation of rs-fMRI Measures

The ALFF measures regional spontaneous neural activity (Zang et al., 2007). Each preprocessed fMRI data set was transformed to a frequency domain with a fast Fourier transformation. The square root of the power spectrum was calculated, and the ALFF was obtained as the averaged square root across 0.01–0.1 Hz. The ALFF value of each voxel was then divided by the global mean ALFF value for each participant to reduce the global effects. ALFF was computed as the mean power spectrum in a specific low-frequency band (0.01–0.1 Hz; Zang et al., 2007), and the fALFF was the ratio of the power spectrum in the low-frequency band (0.01–0.1 Hz) to the entire frequency range (Zou et al., 2008). The fALFF value of each voxel was then divided by the global mean fALFF value for each participant to reduce the global effects.

The DC measures the mean correlation between a given region of interest (ROI) and all other ROIs in the functional brain network (Zang et al., 2004). An ROI with a higher DC value suggests that it is more functionally connected with other ROIs than one with a lower DC value. In graph theory, DC is defined as the number (binary graph) or the sum of weights (weighted graph) of edges connecting to a node. Here, we computed Pearson’s correlation coefficients between the BOLD time courses of all pairs of voxels and obtained a whole gray matter functional connectivity matrix for each participant. For a given voxel, DC was computed as the sum of positive functional connectivity above a threshold of 0.25 between that voxel and all other voxels within the gray matter (Buckner et al., 2009; Zuo et al., 2012). The individual level DC map was converted into a z-score map by subtracting the mean of the brain mask DC and dividing by the standard deviation of the whole brain mask DC.

### Statistical Analysis

To investigate the differences in the demographics and clinical characteristics of the patients, pre/post-treatment, the paired sample t-test was used for continuous variables. The paired sample t-test was performed in SPM12 to examine differences in ALFF, fALFF, and DC between participants pre/post-treatment. All other statistical analyses were conducted using SPSS (version 25.0; IBM, Armonk, NY, USA) with a statistical significance of *P* < 0.05 [false discovery rate (FDR) corrected].

At baseline, Pearson correlation analyses were performed to examine the correlations between the mean value of measures in the brain regions showing significant differences and clinical symptoms (HAMD/BSSI). After treatment, Pearson correlation analyses were used to examine whether the changes of these measures were correlated with the changes in clinical symptoms. Changes in HAMD (ΔHAMD) and BSSI (ΔBSSI) were calculated using the following equations:


$$\Delta \text{HAMD}=\frac{\text{preHAMD}-\text{postHAMD}}{\text{preHAMD}}$$

$$\Delta \text{BSSI}=\frac{\text{preBSSI}-\text{postBSSI}}{\text{preBSSI}}$$

Pearson correlation analyses were also used to examine whether these measures at baseline were correlated with changes in clinical symptoms (ΔHAMD/ΔBSSI).

## Results

The psychological measurements and demographic data are listed in Table 1. Compared to post-treatment, participants pre-treatment demonstrated more severe symptoms according to HAMD and BSSI scores. There were significant improvements in HAMD scores (*P* < 0.001) and BSSI scores (*P* < 0.001; Table 1).

**Table 1:** Demographics and clinical characteristics pre/post-treatment.

Characteristic	Pre-treatment (*n* = 14)	Post-treatment (*n* = 14)	*P*
Age, mean (SD), y	14.57 (1.45)	/	
Sex (Male/Female)	5/9	5/9	
Education years, mean (SD), y	8.35 (1.39)	/	
HAMD, mean (SD)	30.14 (3.78)	11.36 (3.13)	<0.001
BSSI, mean (SD)	21.43 (3.67)	6.93 (3.58)	<0.001

*Note: HAMD, Hamilton depression scale; BSSI, Beck scale for suicide ideation; SD, Standard deviation*.

**Figure 1:**
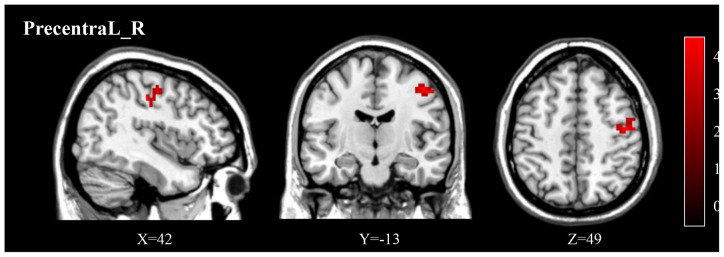
The post-treatment adolescents with MDD exhibited a significantly decreased ALFF in the right precentral gyrus (Precentral_R). ALFF, amplitude of low frequency fluctuation; MDD, Major depressive disorder.

**Figure 2:**
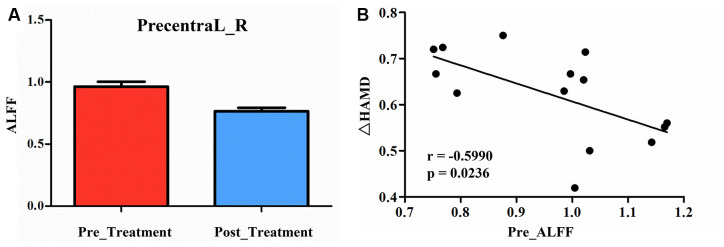
**(A)** Post-treatment, adolescents exhibited a significantly decreased ALFF in the right precentral gyrus (Precentral_R). **(B)** The negative correlations between ΔHAMD and ALFF of the Precentral_R at baseline. HAMD, Hamilton depression scale.

**Figure 3:**
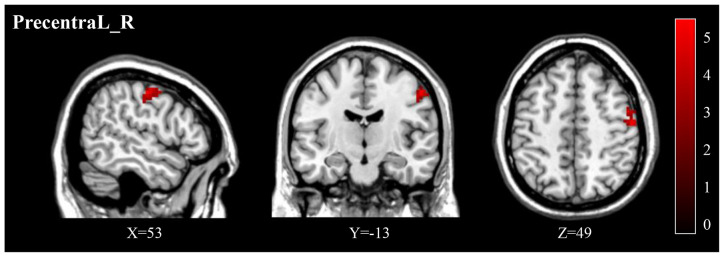
Post-treatment, adolescents exhibited a significantly decreased fALFF in the right precentral gyrus (Precentral_R). fALFF, fractional ALFF.

**Figure 4:**
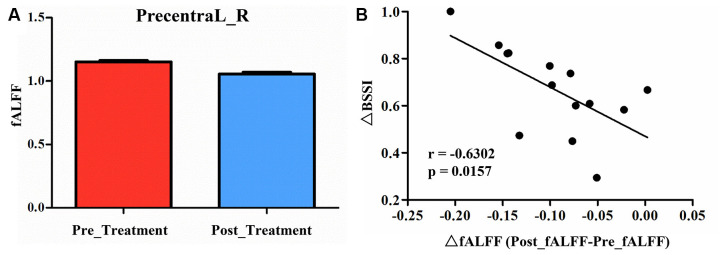
**(A)** Post-treatment, adolescents exhibited a significantly decreased fALFF in the right precentral gyrus (Precentral_R). **(B)** The negative correlations between the ΔBSSI and ΔfALFF in the Precentral_R.

**Figure 5:**
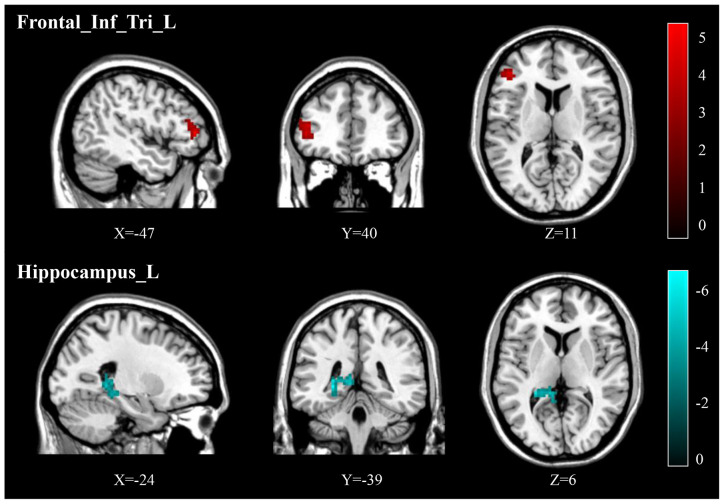
Post-treatment, adolescents exhibited decreased DC in the triangular part of the left inferior frontal gyrus (Frontal_Inf_Tri_L) and increased DC in the left hippocampus (Hippocampus_L).

**Figure 6:**
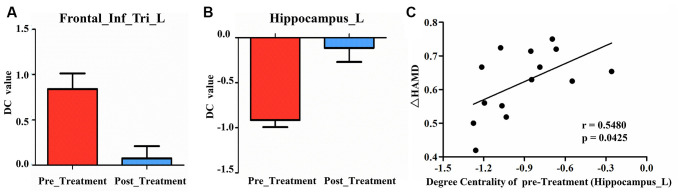
**(A)** Post-treatment, adolescents exhibited decreased DC in the triangular part of the left inferior frontal gyrus (Frontal_Inf_Tri_L). **(B)** Post-treatment, adolescents exhibited increased DC in the left hippocampus (Hippocampus_L). **(C)** Our correlation analysis showed that ΔHAMD was positively correlated with the DC value of Hippocampus_L at baseline.

Compared to pre-treatment, adolescents post-treatment exhibited decreased ALFF and fALFF in the right precentral gyrus (Precentral_R; Figures 1, 2A, 3, 4A; Table 2). Moreover, post-treatment, decreased DC values were found in the left triangular part of the inferior frontal gyrus (Frontal_Inf_Tri_L) and increased DC values in the left hippocampus (Hippocampus_L; Figures 5, 6A,B; Table 2).

**Table 2:** Significant differences in ALFF, fALFF, and DC between depression adolescents pre/post-treatment.

**Measures**	**Brain regions**	**Voxel size**	**Peak T value**	**MNI coordinates**		
**Decreased**
ALFF	PrecentraL_R	71	4.40	46	−12	46
fALFF	PrecentraL_R	52	5.10	51	−9	51
DC	Frontal_Inf_Tri_L	70	5.65	−45	42	6
**Increased**
DC	Hippocampus_L	105	6.40	−27	−39	0

*Note: DC, degree centrality; MNI, Montreal Neurological Institute*.

We found significantly negative correlations between the ΔHAMD and ALFF of the Precentral_R at baseline (*r* = −0.5990, *P* = 0.0236; Figure 2B) and between the ΔBSSI and the change of fALFF in the Precentral_R (*r* = −0.6302, *P* = 0.0157; Figure 4B). In addition, correlation analysis demonstrated that ΔHAMD was positively correlated with the DC value of the Hippocampus_L at baseline (*r* = 0.5480, *P* = 0.0425; Figure 6C).

## Discussion

In our present study, in a sample of adolescent patients with MDD and SI, the severity of both MDD and SI substantially decreased after 2 weeks of ECT. Previous studies have found that 2 weeks of repetitive transcranial magnetic stimulation could decrease HAMD scores significantly in adults with increased regional function in the left dorsolateral prefrontal cortex (Zheng et al., 2020), but did not discuss SI. Shen et al. (2015) found that 2 weeks of pharmacological therapy could alter DC in the middle frontal gyrus and precuneus; however, this study also lacked focus on SI. Post-ECT, our present study found changed brain function in the precentral gyrus, hippocampus, and the triangular part of the inferior frontal gyrus, which may indicate the mechanism of action behind the efficacy of ECT in adolescents with MDD and SI.

Studies have demonstrated that the hippocampus has been linked to mood disorders that begin during adolescence, which show major cognitive and emotional disturbances (Masi and Brovedani, 2011; Hueston et al., 2017). Other previous studies have found that ECT induces structural changes in the hippocampus (Redlich et al., 2016; Sartorius et al., 2016). The most consistent finding from previous studies on the hippocampus depicted substantial reductions in hippocampal volume in MDD patients compared to healthy controls, which increased following ECT (Boccia et al., 2015; Arnone et al., 2016; Peng et al., 2016). Tendolkar et al. (2013) found bilateral volume increases in the hippocampus after ECT, which was further verified to occur in the right hippocampus by Abbott et al. (2014). Smaller hippocampal volumes were found in patients with MDD and a history of SA compared to those without SA (Colle et al., 2015). Therefore, this may indicate that a potential mechanism underlying ECT in MDD is through the increase in hippocampal volume.

Additional previous studies have found that DC differed between in psychiatric disorders; One study demonstrated that DC changes in the hippocampus are related to delayed encephalopathy after carbon monoxide poisoning (DEACMP). Compared with healthy controls, DEACMP patients with cognition disturbances displayed significantly decreased DC values in the right hippocampus but increased DC values in the right inferior frontal gyrus, which is inconsistent with our results (Wu et al., 2020). This inconsistency may be related to differences in characteristics or diseases, but can also indicate that abnormal brain function in the hippocampus may present as changed DC. An additional study found that decreased DC values in the frontoparietal network could distinguish patients who had experienced SA from those with SI, but it focused on adults and lacked longitudinal data (Wagner et al., 2021). A 2-week pharmacological therapy for MDD patients found correlations in baseline DC with changes in the HAMD scores, including in the precuneus, supramarginal gyrus, middle temporal, but not in the hippocampus, this might be due to different treatment compared with our study (Shen et al., 2015). In the present study, we also found decreased DC in the inferior frontal gyrus post-ECT, which was consistent with previous studies demonstrating that patients with past SA had abnormal brain activity and DC values were found in the inferior frontal gyrus (Makris et al., 2007; Wagner et al., 2021). Wu et al. (2021) found patients with MDD showed abnormal DC in the prefrontal cortex (PFC), and the DC of PFC was negatively correlated with the course of the disease, not with the HAMD scores, however, these results should be interpreted cautiously with ours. Therefore, further studies are needed.

Previous studies found that patients with MDD have altered ALFF/fALFF in various regions, such as the precentral gyrus. Wang et al. (2012) found that patients with MDD demonstrated increased ALFF in the right fusiform gyrus, but no change in the precentral gyrus; however, the fALFF in patients was significantly increased in the right precentral gyrus compared to healthy controls. For MDD patients with SI, Chen et al. (2021) found higher mfALFF of the right middle temporal pole gyrus in the SI group compared with the NS group, similar results were not found in the precentral gyrus, but a positive correlation between depression score and mfALFF was found in the right postcentral gyrus, showed high HAMD scores correlated with higher mfALFF, which was similar with our study. Kong et al. (2017) found that older patients with MDD administered ECT demonstrated decreased ALFF values in the precentral gyrus compared to pre-treatment, which is consistent with our results. We suggest that lower ALFF/fALFF in the precentral gyrus may indicate better outcomes for patients with MDD.

Several studies have focused on the changes of ALFF/fALFF in adolescents with MDD pre/post-treatment, with treatment options being pharmacotherapy, psychotherapy, or pharmacotherapy combined with psychotherapy. Kim et al. (2018) found behavioral difficulties in adolescent bullies with depressed mood, and after cognitive behavioral therapy (CBT), decreased fALFF in the inferior parietal lobule and the lingual, interior frontal, and middle occipital gyri were demonstrated. Shu et al. (2020) found that in young patients with MDD and SA after CBT and antidepressant co-therapy, fALFF in the left middle occipital cortex and left precuneus were significantly increased in the CBT group compared with the healthy control group. Fan et al. (2013) found SA patients had increased ALFF in the right superior temporal gyrus relative to non-suicidal patients. Cao et al. (2016) found the SA group showed increased zALFF in the right superior temporal, left middle temporal, and left middle occipital gyri in young patients with MDD aged 15–29 years, but additional research is lacking.

These aforementioned studies are only partly consistent with our current research. This may be related to different treatment options, methods of patient evaluation, the severity of disease, and diagnosis. Notably, it can be difficult to distinguish unipolar from bipolar depression in adolescents. There are limited studies that aim to understand the efficacy of ECT using fMRI in adolescents with MDD and SI; our results suggest that changes in ALFF/fALFF and DC after ECT in various brain regions may be a potential mechanism behind the efficacy of ECT in adolescents with MDD and SI.

### Limitations

Several limitations of our current study should be noted. First, our sample size was small, possibly due to concerns about ECT side effects. Second, there was a lack of healthy controls to compare the brain function with the patient group at baseline; therefore, further research is needed.

## Conclusion

We found decreased ALFF/fALFF in the right precentral gyrus, decreased DC in the triangular part of the left inferior frontal gyrus and increased DC in the left hippocampus in adolescents with MDD and SI after ECT. ALFF in the right precentral gyrus at baseline and DC in the left hippocampus at baseline, changes in fALFF in the right precentral gyrus were correlated with treatment outcome. We suggest that these brain regions may be potential indicators of ECT response in adolescents with MDD and SI.

## Data Availability Statement

The raw data supporting the conclusions of this article will be made available by the authors, without undue reservation.

## Ethics Statement

The studies involving human participants were reviewed and approved by Human Research and Ethics Committee of the First Affiliated Hospital of Chongqing Medical University (no. 2017-157). Written informed consent to participate in this study was provided by the participants’ legal guardian/next of kin.

## Author Contributions

XL conceived the structure of the manuscript and wrote the manuscript. RY and QH prepared the samples and did fMRI. XC, YZ, LD, and XQ analyzed the data. MA and LK critically reviewed the manuscript. All authors have read and approved the final manuscript. All authors contributed to the article and approved the submitted version.

## Conflict of Interest

The authors declare that the research was conducted in the absence of any commercial or financial relationships that could be construed as a potential conflict of interest.

## Publisher’s Note

All claims expressed in this article are solely those of the authors and do not necessarily represent those of their affiliated organizations, or those of the publisher, the editors and the reviewers. Any product that may be evaluated in this article, or claim that may be made by its manufacturer, is not guaranteed or endorsed by the publisher.

## Footnotes

^1^ http://www.restfmri.net

^2^ http://www.fil.ion.ucl.ac.uk/spm
